# Molecular Detection of Antibiotic Resistance Genes Using Respiratory Sample from Pneumonia Patients

**DOI:** 10.3390/antibiotics14050502

**Published:** 2025-05-13

**Authors:** Eman Abdullah Alsuof, Ahmad R. Alsayed, Manar Saleh Zraikat, Heba A. Khader, Luai Z. Hasoun, Mamoon Zihlif, Osama Abu Ata, Malek A. Zihlif, Mahmoud Abu-Samak, Mohammed Al Maqbali

**Affiliations:** 1 Department of Pharmacology, School of Medicine, The University of Jordan, Amman 11942, Jordan; 2 Department of Clinical Pharmacy and Therapeutics, Faculty of Pharmacy, Applied Science Private University, Amman 11931, Jordan; 3 Department of Clinical Pharmacy and Pharmacy Practice, Faculty of Pharmaceutical Sciences, The Hashemite University, Zarqa 13133, Jordan; 4 Department of Internal Medicine, Section of Pulmonary Diseases, Islamic Hospital, Amman 11181, Jordan; mamoonz@hotmail.com; 5 Department of Internal Medicine, Section of Infectious Disease, Islamic Hospital, Amman 11181, Jordan; osamaabuata@yahoo.com; 6 Department of Internal Medicine, Fatima College of Health Sciences, Al Ain 999041, United Arab Emirates

**Keywords:** antimicrobial, antibiotic-resistant genes, hospital, epidemiology, pneumonia

## Abstract

Introduction/Objectives: Antibiotic resistance makes the treatment of pneumonia challenging. Effective management depends on accurate diagnostic techniques to identify resistance genes and customize drugs. This study primarily aimed to identify antibiotic resistance genes in respiratory samples from patients with pneumonia using polymerase chain reaction (PCR) to determine the prevalence of specific resistance genes and analyze clinical factors contributing to antibiotic resistance, as well as to provide actionable insights into resistance patterns in Jordan and support efforts to improve pneumonia management. Methods: This retrospective observational study included 114 patients who were diagnosed with pneumonia. Clinical data, including prior antibiotic exposure and treatment history, were collected. PCR diagnostics were used to detect resistance genes in respiratory samples. In this study, we evaluated 14 antibiotic resistance genes in pneumonia pathogens, highlighting their diverse resistance mechanisms. Results: *Mec A* was the most frequently detected gene, appearing in 87 samples (77.3%). Additionally, *Tem* in 80 samples (70.2%), *Oxa-48-like* in 15 samples (13.2%), and *Ctx-M-1* in 38 samples (33.3%) were among the most commonly detected genes. In contrast, *Oxa-40-like* (7.0%), *Vim* (8.8%), and *Imp* (4.4%) genes exhibited a lower prevalence. The *Oxa-51-like* gene showed the only significant association with ertapenem resistance (*p*-value = 0.046). Further analysis revealed statistically significant associations between *Mec A* and methicillin resistance (*p* < 0.001), underscoring its critical role. However, other genes, such as *Oxa-40-like* and *Oxa-48-like*, showed no significant correlation with the antibiotic resistance patterns of imipenem and meropenem (*p* > 0.05). Conclusions: This study demonstrates the utility of PCR-based diagnostics for detecting resistance genes and highlights the critical clinical factors associated with antibiotic resistance in patients with pneumonia. These findings underscore the importance of integrating molecular diagnostics into routine care to improve treatment outcomes and combat the growing threat of antibiotic resistance in Jordan. This highlights PCR’s value in guiding effective treatment strategies and addressing multidrug-resistant pneumonia.

## 1. Introduction

Antibiotic resistance is a global health crisis with far-reaching implications. The overuse and misuse of antibiotics have accelerated the emergence of resistant bacterial strains, making it increasingly difficult to treat infections that are easily managed [1,2]. This challenge is particularly acute in the case of pneumonia, where rapid and accurate detection of antibiotic resistance is crucial for effective treatment. The rise of multidrug-resistant pathogens has led to longer hospital stays, increased medical costs, and higher mortality rates, underscoring the urgent need for early and precise diagnostic methods to identify resistant strains and guide appropriate therapies [3].

Pneumonia remains one of the leading causes of morbidity and mortality worldwide, affecting millions of people annually. In Jordan, the burden of pneumonia is significant, particularly among vulnerable populations, such as the elderly and those with underlying health conditions. Timely and effective treatment is essential for preventing complications and reducing mortality. However, the increasing prevalence of antibiotic-resistant bacteria complicates treatment efforts, making it challenging to achieve positive patient outcomes [4].

Various diagnostic techniques have been employed to identify the causative pathogens of pneumonia, ranging from traditional microbiological culture to advanced molecular methods [5,6]. Although culture methods are considered the gold standard, they are time-consuming and can delay the initiation of appropriate therapy [7]. In contrast, molecular techniques such as polymerase chain reaction (PCR) offer rapid and highly sensitive detection of pathogens and antibiotic-resistance genes, enabling quicker clinical decision-making. PCR has emerged as a powerful tool for the diagnosis of pneumonia, especially in cases where rapid identification of resistant strains is crucial [8].

The growing threat of antibiotic resistance in pneumonia-causing pathogens presents a significant public health challenge. With the rise of multidrug-resistant strains, there is a pressing need for diagnostic methods that can quickly and accurately identify resistant pathogens [9].

The primary objective of this study was to detect antibiotic resistance genes in sputum and bronchoalveolar lavage (BAL) samples obtained from patients with pneumonia using PCR. In addition, this study aimed to evaluate the prevalence of specific resistance genes and assess the clinical and demographic factors associated with antibiotic resistance in patients with pneumonia. These findings are expected to provide valuable insights into the prevalence of antibiotic resistance in Jordan and help refine diagnostic practices to enhance patient outcomes in the treatment of pneumonia.

This study aimed to address this need by developing and validating PCR-based diagnostic techniques that can be integrated into routine clinical practice. By focusing on the detection of antibiotic resistance genes in sputum and BAL samples from patients with pneumonia, this study seeks to improve the management of pneumonia in Jordan and contribute to the global effort to combat antibiotic resistance.

## 2. Methods and Materials

### 2.1. Study Framework

This retrospective observational study aimed to identify the antibiotic resistance genes in bacterial strains isolated from patients with pneumonia. Samples were collected in 2022 from 114 patients at the Islamic Hospital. Table 1 summarizes the key aspects of the study design.

### 2.2. Sample Acquisition

Participants were selected based on the inclusion and exclusion criteria. Cases without a pneumonia diagnosis, insufficient samples, or contaminated samples were excluded. Detailed criteria are listed in Table 2.

### 2.3. Sample Types

The sputum (SPU) was the most frequently collected sample type. The other sample types included the following:

- Fluid samples.
- Bronchoalveolar lavage (BAL).
- Endotracheal aspirates.

### 2.4. Sample Handling and Storage

Immediate Processing: Samples were promptly transported to the laboratory and stored at −80 °C to preserve the integrity of the genetic material until processing [10,11].

### 2.5. Patient Demographics and Clinical Characteristics

The following table summarizes the key demographic and clinical characteristics of the patients included in the study. It includes data on sex, age, prior antibiotic use, and clinical features, such as respiratory distress and hemoptysis. The CURB-65 score was used to assess pneumonia severity, while microbiological data, including culture results, were used as clinical data for pathogen identification (Table 3).

### 2.6. Genetic Material Extraction

Several key reagents and materials were used for DNA extraction, each serving a specific function to ensure the efficient extraction and purification of DNA. Table 4 summarizes the reagents and their specific purposes in the DNA extraction process.

Table 5 summarizes the materials and methods used for bacterial DNA extraction from lower respiratory tract samples. Bacterial DNA extraction was performed using the ZymoBIOMICS™ DNA Miniprep Kit (Zymo Research, Tustin, CA, USA) according to the manufacturer’s instructions, with 200 µL of the sample processed per the kit protocol.

The key steps include cell lysis, DNA binding, washing, and elution. The process aims to efficiently extract high-quality DNA suitable for downstream applications, including PCR [12,13].

### 2.7. Amplification via PCR

#### 2.7.1. Diagnostic Tool: BacResista GLA REAL-TIME PCR Detection Kit

In this study, we used the BacResista GLA REAL-TIME PCR Detection Kit for the rapid molecular detection of some of the most important antibiotic resistance genes, which could allow the precise identification of resistance in different critical bacteria. This commercial diagnostic assay was designed to directly detect resistance determinants from DNA extracted from clinical respiratory specimens or bacteria isolated from these specimens. The key feature of the kit is the detection of resistance to glycopeptide (G)-, beta-lactam (L)-antibiotics, and carbapenems (A), which represent three major antimicrobial classes encountered in clinical practice.

The detection panel of this multiplex PCR assay is composed of the widest range of targets described for any healthcare-associated pathogen, including genes encoding resistance to glycopeptides (*VanA*, *VanB*), beta-lactams (*MecA*, *Tem*, *SHV*, *CTX-M-1*), and carbapenems (*OXA-23-like*, *OXA-40-like*, *OXA-48-like*, *OXA-51-like*, *IMP*, *KPC*, *GES*, *NDM*, and *VIM*). These genes encompass many of the most important clinical resistance mechanisms found in clinical settings (including methicillin-resistant Staphylococcus aureus (MRSA), extended-spectrum beta-lactamase (ESBL), producing *Enterobacteriaceae* and carbapenem-resistant *Acinetobacter* and *Klebsiella*), and also mediate resistance to several clinically important antibiotic classes.

The resistance genes were detected, and their corresponding antibiotics are listed in Table 6. These biomarkers are important for directed therapy, especially in critically ill patients in whom inadequate empirical treatment can result in poor prognosis.

Multiplex real-time PCR technology used in this assay to amplify and detect multiple gene targets in one reaction tube plays an important role in improving the efficiency of diagnosis. This molecular test can provide results in as little as a few hours, whereas standard culture and susceptibility testing can take up to 72 h. Early and appropriate targeted antibiotic therapy, antimicrobial stewardship, and control of the spread of resistance genes in healthcare settings all depend on a short time to result.

Polymerase chain reaction (PCR) was performed to amplify target DNA sequences. Table 7 outlines the materials and steps involved in PCR.

#### 2.7.2. Detection Channels of Amplification Products

Subsequent to bacterial DNA extraction, each sample was screened for antibiotic resistance genes using the BACRESISTA GLA REAL-TIME PCR Detection Kit (DNA Technology, Moscow, Russia). A Ct value ≥ 45 was considered not detected, and a Ct value < 45 was considered detected. Table 8 lists specific target genes and their corresponding detection channels.

#### 2.7.3. Procedure of PCR Amplification

The Taq-polymerase solution was vortexed for 3–5 s and then briefly spun for 1–3 s to collect drops. Next, 10 µL of Taq-polymerase solution was added to each strip tube, taking care to avoid breaking the paraffin layer. A drop of approximately 20 µL of mineral oil was added to each strip tube, and the tubes were closed. Tubes containing DNA samples, positive control samples, and negative control samples were vortexed for 3–5 s, followed by a brief spin for 1–3 s to ensure that all drops were collected.

Next, 5.0 µL of the DNA sample was added to the corresponding strips or tubes, making sure not to add DNA to the “C-” or “C+” strip tubes. Care was taken to avoid the breakage of the paraffin layer. Next, 5.0 µL of the negative control (C-) that had passed the entire DNA extraction procedure was added to the “C-” tube, while 5.0 µL of the positive control (C+) was added to the corresponding strip or tube. The strips or tubes were then spun for 1–3 s to collect drops. Finally, the strips or tubes were placed in a Real-time Thermal Cycler.

The operating software was then launched. The corresponding test was added, and the number and IDs of the samples, along with the positive and negative control samples, were specified. The positions of the strips or tubes in the thermal unit were assigned. PCR was then initiated, and the process was run as shown in Table 9. The amplification program for target detection was executed on a Quant Gene 9600 instrument (BIOER).

## 3. Results

The findings of this study, including demographic and clinical features, hospitalization characteristics, clinical outcomes, microbial pathogenesis, clinical features, risk factors, and pneumonia severity, are summarized in Table 10. A comprehensive overview of these results is provided in Table 11, which consolidates the key data points for clarity and ease of referencing.

### 3.1. Demographic and Clinical Features

In total, 114 patients with community-acquired pneumonia (CAP) were included in this study. The study had a median age of 73 years (IQR: 47–79 years) and a mean age of 64.114 years (±20.331), reflecting a predominantly elderly population. The youngest patient was 16 years old, whereas the oldest was 95 years old. Male patients constituted 55.263% (n = 63), whereas females accounted for 44.737% (n = 51). This gender distribution aligns with global data on pneumonia prevalence, where males are typically more affected due to higher risk factors, such as smoking and chronic lung diseases.

#### 3.1.1. Length of Stay and Hospitalization Characteristics

The median length of hospital stay (LOS) was 7 days (IQR: 3–10), with a mean of 11.272 days (±15.091). Hospital stays ranged from a minimum of one day to a maximum of 83 days, reflecting significant variability in disease severity. Among patients requiring ICU admission, the median ICU length of stay was 1 d (IQR: 0–5), with a mean of 4.763 days (±11.882). The median number of hospitalizations was 2 (IQR: 1–3) with a mean of 2.772 (±3.005), and the median number of pneumonia episodes was 0.5 (IQR: 0–1) with a mean of 0.895 (±1.366).

#### 3.1.2. Clinical Outcomes

Of the 114 patients, 23.684% (n = 27) succumbed to their illness, indicating a high mortality rate consistent with severe pneumonia in older populations. Conversely, 76.316% (n = 87) of patients showed clinical improvement and were successfully discharged. This mortality rate highlights the burden of CAP in vulnerable populations, particularly in the presence of comorbidities or antibiotic resistance.

#### 3.1.3. Microbial Pathogenesis

Microbiological analysis has revealed diverse etiologies of CAP, with bacterial pathogens being the most frequently identified. Bacterial infections accounted for 28.947% (n = 33) of the cases, followed by bacterial-viral co-infections (24.561%, n = 28). Viral infections alone constituted 20.175% (n = 23) of the cases, and polymicrobial infections were common, including polybacterial (10.526%, n = 12) and polymicrobial viral infections (13.158%, n = 15). Fungal-bacterial co-infections were the least frequent, comprising 1.754% (n = 2). These findings emphasize the complexity of CAP pathogenesis, with a significant proportion of cases involving mixed infections, which may complicate the treatment strategies. The data were obtained from another study in which PCR tests were performed on these samples.

#### 3.1.4. Clinical Features and Risk Factors

The most common clinical features in this study included shortness of breath (SOB), which was observed in 54.369% (n = 56) of the patients, and cough, which occurred in 13.158% (n = 15). Fever was noted in 13.592% (n = 14) of cases, while sputum production was recorded in 6.140% (n = 7). Less frequent symptoms included pleuritic chest pain (8.738%; n = 9). Hemoptysis or rust-colored sputum was absent in 103 patients (100%) due to 11 missing values, suggesting that none of the patients with available data exhibited these symptoms.

Regarding comorbidities, COPD was present in 3.509% (n = 4) of the patients, and asthma was documented in 5.263% (n = 6). A significant majority (91.228%, n = 104) of the patients had not received immunosuppressive treatment, indicating that most individuals in this study did not have this specific risk factor.

In terms of respiratory distress, 7.018% (n = 8) of the patients demonstrated signs of respiratory distress, while 84.211% (n = 96) did not exhibit these signs. The prevalence of aspiration pneumonia was 15.358% (n = 16), and 5.769% (n = 6) of the patients required intubation with mechanical ventilation, reflecting the severity of pneumonia in these cases.

Regarding prior antibiotic use, 91.228% (n = 104) of patients did not have any antibiotic exposure within 30 days preceding their current illness. This high proportion suggests that recent antibiotic use may not be a significant factor in the selection of antibiotic-resistant strains in this population.

#### 3.1.5. Severity and Guideline Adherence

The CURB-65 score, used to assess pneumonia severity, showed that 24.561% (n = 28) of patients had a score of 0 and 57.895% (n = 66) had a score of 1, indicating low-risk CAP. A detailed summary of pneumonia severity is provided in Table 11 for a comprehensive overview.

The PSI (pneumonia severity index), also known as the PORT score, was used to assess the severity of pneumonia and to predict patient outcomes. Based on PSI scores, patients were categorized into four risk classes, with each class corresponding to specific mortality rates and clinical management strategies.

- Class I (Low Risk, PSI 0–50):

A total of 59.649% (n = 68) of patients had a PSI score ≤50, indicating a low mortality risk (0.1%) and were typically managed with outpatient care. This class represented the largest proportion of the studies.

- Class II (Low to Moderate Risk, PSI 51–70):

A total of 24.561% (n = 28) of the patients had PSI scores between 51 and 70, placing them at low to moderate risk, with a mortality rate of 0.6%. These patients may require either outpatient care or observation admission, depending on their clinical judgment.

- Class III (Moderate Risk, PSI 71–90):

A total of 8.772% (n = 10) of the patients had PSI scores between 71 and 90, indicating moderate risk with a mortality rate of 0.9%. Inpatient admission is generally recommended for these patients because of the potential for complications and the need for closer monitoring.

- Class IV (High Risk, PSI > 130):

A total of 7.018% (n = 8) of patients had PSI scores > 130, classifying them as high-risk with a mortality rate of 9.3%. These patients typically require inpatient admission and intensive management because of their significantly elevated risk of adverse outcomes.

Guideline-concordant antibiotic use was recorded in only 35.106% (n = 33) of the cases, underscoring suboptimal adherence to treatment protocols. Completion of the prescribed antibiotic therapy was achieved in 63.158% (n = 72) of patients, while 36.842% (n = 42) either discontinued treatment prematurely or received incomplete regimens.

### 3.2. Antibiotic Sensitivity Test

**Figure 1:**
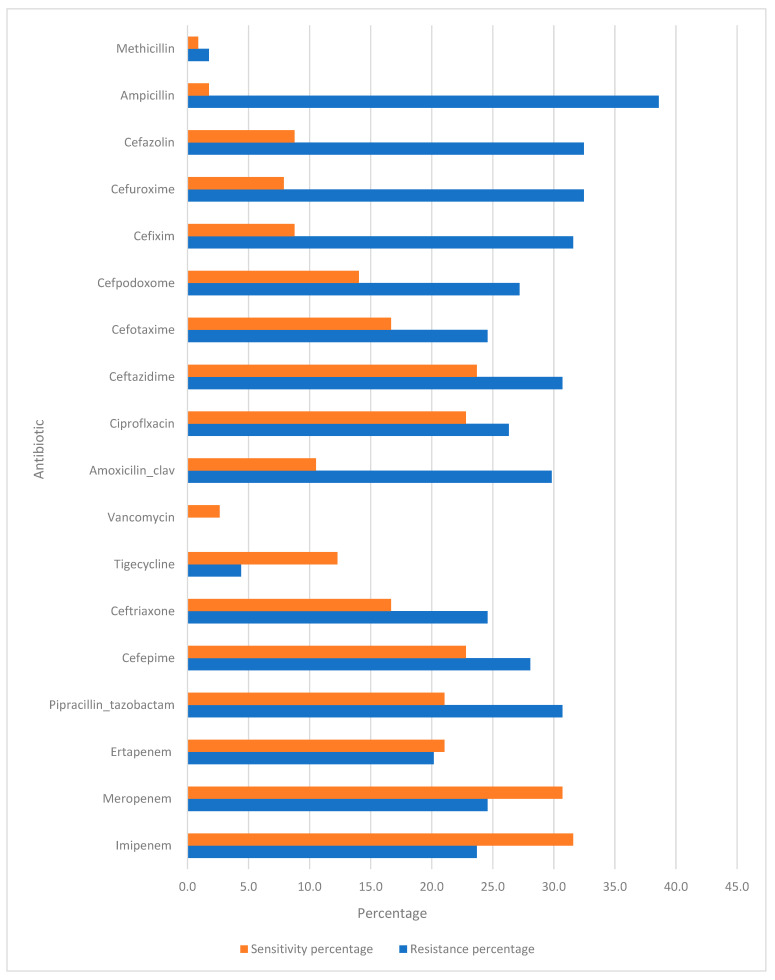
Antibiotic sensitivity test.

The management of community-acquired pneumonia (CAP) is increasingly challenged by the rising incidence of antibiotic resistance. The data provided in Figure 1 highlight the resistance and sensitivity patterns of various antibiotics used. Understanding these patterns is crucial for developing effective treatment strategies.

**Imipenem and Meropenem:** Both carbapenems, Imipenem and Meropenem, exhibit resistance rates of 23.7% and 24.6%, respectively. The corresponding sensitivity percentages were 31.6% and 30.7%.

**Ertapenem:** This carbapenem showed a slightly lower resistance rate (20.2%), with a sensitivity of 21.1%.

**Piperacillin–Tazobactam:** With a resistance rate of 30.7% and sensitivity of 21.1%, this combination antibiotic is frequently used for its synergistic effect against a wide range of pathogens.

**Cefepime, Ceftriaxone, and Ceftazidime:** These cephalosporins showed varying resistance rates (28.1%, 24.6%, and 30.7%, respectively) and sensitivities (22.8%, 16.7%, and 23.7%, respectively). Ceftriaxone and Ceftazidime are often used as first-line agents for CAP because of their efficacy against *Streptococcus pneumoniae* and *Haemophilus influenzae.* Cefepime is typically reserved for more severe cases or suspected Gram-negative infections owing to its extended spectrum.

**Tigecycline:** This antibiotic has a notably low resistance rate of 4.4% and sensitivity of 12.3%.

**Amoxicillin–Clavulanate and Ciprofloxacin:** Amoxicillin–Clavulanate showed a resistance rate of 29.8% and a sensitivity of 10.5%, whereas ciprofloxacin had a resistance rate of 26.3% and a sensitivity of 22.8%. Both antibiotics are commonly used for outpatient treatment of CAP.

**Cefotaxime, Cefpodoxime, Cefixime, Cefuroxime, and Cefazolin:** These cephalosporins exhibited significant resistance rates ranging from 24.6% to 32.5%, with sensitivities between 7.9% and 16.7%. Their use in CAP treatment is often guided by the susceptibility results.

**Ampicillin and Methicillin:** Ampicillin showed a high resistance rate of 38.6% with a minimal sensitivity of 1.8%, reflecting limited efficacy against common CAP pathogens. The exceptionally low resistance (1.8%) and sensitivity (0.9%) confirmed its limited role, which is primarily of historical interest.

### 3.3. Antibiotic Utilization Patterns

The antibiotic utilization patterns in this study reflect the use of empirical and subsequent therapies. The findings are summarized in Table 12 which provides a detailed breakdown of empiric therapy, next-line therapy, and initial continuation therapy.

### 3.4. Empiric and Subsequent Antibiotic Use

#### 3.4.1. Empiric Therapy

Empiric therapy was prescribed to most patients (n = 114), with **meropenem** being the most frequently used antibiotic, accounting for 31.60% (n = 36) of cases. **Piperacillin–tazobactam** was the second most common empiric antibiotic used in 26.30% (n = 30) of cases. Other antibiotics included **amoxicillin–clavulanate** (7.90%, n = 9), which was favored for moderate infections, and **ceftriaxone** (6.10%, n = 7), which is often prescribed for community-acquired infections. The less frequently used antibiotics included **levofloxacin** (3.50%, n = 4) for respiratory pathogens, **azithromycin** (4.40%, n = 5) for atypical coverage, and **ampicillin** (3.50%, n = 4) for Gram-positive infections. **Vancomycin** was prescribed in 23.70% (n = 27) of cases, reflecting its role in managing suspected MRSA infections. Targeted therapies such as **doxycycline** (0.90%, n = 1) and **amikacin** (1.80%, n = 2) were sparingly used for atypical and Gram-negative pathogens, respectively.

#### 3.4.2. Next-Line Therapy

Next-line therapies were initiated in patients who did not respond to empirical treatment or required escalation due to multidrug-resistant organisms. Amoxicillin–clavulanate was the most commonly used next-line antibiotic, prescribed in 14.04% (n = 16) of cases to narrow down therapy when pathogens were susceptible. Azithromycin and colistin were each used in 9.64% (n = 11) of the cases, with colistin targeting multidrug-resistant Gram-negative pathogens and azithromycin providing coverage for respiratory and atypical pathogens.

#### 3.4.3. Initial Continuation Therapy

Empiric antibiotics were continued as the initial therapy in some cases when clinical progress or culture results supported their use. Colistin was the most frequently continued antibiotic, used in 11.40% (n = 13) of the cases, primarily for resistant Gram-negative organisms. Doxycycline was continued in 7.02% (n = 8) of cases of atypical pathogens, whereas azithromycin was continued in 6.14% (n = 7) of patients with respiratory or atypical infections. Other antibiotics included amikacin (4.39%, n = 5), metronidazole (1.75%, n = 2) for anaerobic infections, levofloxacin (2.63%, n = 3), and amoxicillin–clavulanate (0.88%, n = 1). Ceftriaxone was continued in 0.88% (n = 1) of the cases when susceptibility testing confirmed its efficacy.

The hospital opted for carbapenems as empiric therapy for patients with CAP due to their broad-spectrum activity against a wide range of pathogens, including resistant strains. Carbapenems, particularly meropenem, are effective against Gram-negative bacteria that are commonly involved in severe CAP, such as those caused by multidrug-resistant organisms. Their strong effectiveness against organisms, such as *Escherichia coli* and *Klebsiella pneumoniae*, which frequently exhibit resistance to other antibiotic classes, makes them a reliable choice in critical settings.

Additionally, the rapid deterioration of patients with CAP and the potential for severe complications necessitate the use of powerful antibiotics that can cover a variety of possible pathogens during the initial treatment phase. The increasing prevalence of resistant bacteria calls for aggressive empirical therapy, and carbapenems serve to bridge this gap until susceptibility patterns can be confirmed through microbiological testing. This approach aims not only to ensure immediate treatment efficacy but also to reduce the risk of treatment failure and the associated morbidity and mortality in these patients.

### 3.5. Antibiotic Resistance Gene Detection

This study investigated the prevalence of antibiotic resistance genes in patients with pneumonia, focusing on several classes of antibiotics. The data revealed variable levels of resistance and sensitivity to different antibiotics, as well as significant variation in the detection of specific resistance genes. A detailed overview of all detected resistance genes and their associations is provided in Table 13.

#### 3.5.1. Imipenem Resistance

*Oxa-40-like* gene was detected in 7% of samples from patients resistant to imipenem, and 93% of samples did not exhibit this gene. The *p*-value for this association was 0.915, indicating no significant correlation between *Oxa-40-like* genes and imipenem resistance.

*Oxa-48-like* gene was found in 13.2% of imipenem-resistant patients, with 86.8% of samples undetected. A *p*-value of 0.200 suggests that there is no statistically significant relationship between the presence of this gene and imipenem resistance.

*Oxa-51-like* gene detection was higher, with 74% of the imipenem-resistant samples showing the gene and 64.9% undetected. The *p*-value for this gene was 0.062, which was statistically significant, but the association was not strong enough to conclude a definitive relationship.

*Imp* gene had a detection rate of 4.4% in imipenem-resistant samples, with a *p*-value of 0.209, indicating no significant association.

*Kpc* was detected in only 1.8% of resistant samples, with a *p*-value of 0.535, indicating that *Kpc* does not significantly contribute to imipenem resistance in the study population.

*Ndm* was present in 33.3% of imipenem-resistant samples, and the *p*-value of 0.601 suggests no significant impact on resistance patterns.

*Vim* gene was detected in 8.8% of resistant samples, with a *p*-value of 0.074, indicating a potential but non-significant association.

#### 3.5.2. Meropenem Resistance

Similar to Imipenem, meropenem resistance showed no significant association with the tested genes (*p*-values for *Oxa-40-like*, *Oxa-48-like*, *Imp*, and *Kpc* ranged from 0.200 to 0.930). The *Oxa-51-like* gene exhibited a trend towards significance, with a *p*-value of 0.093, but the association was not strong enough to conclude a definitive relationship. *Vim* was found in 8.8% of meropenem-resistant samples, with 91.2% of samples undetected. The *p*-value of 0.08 shows a potential, but non-significant, association with meropenem resistance.

#### 3.5.3. Ertapenem Resistance

The *Oxa-51-like* gene showed the only significant association with etapenem resistance, with a *p*-value of 0.046, indicating that it may contribute to resistance in a portion of the population. Other genes tested, including *Oxa40-like*, *Oxa-48-like*, and *Imp*, showed no significant association with ertapenem resistance, indicating that they may not play a major role in resistance in this population.

#### 3.5.4. Methicillin Resistance

The *Mec A* gene was detected in 76.3% of samples resistant to methicillin, with a highly significant *p*-value of 0.000, indicating a strong association between *Mec A* and methicillin resistance.

#### 3.5.5. Resistance to Other Antibiotics

Pipracillin-tazobactam resistance was linked to the *Tem* gene (70.2% of resistant samples), but no statistically significant association was found (*p* = 0.904).

Cefepime resistance also showed a strong association with *Tem* (70.2% resistance), but this was not statistically significant (*p* = 0.848). The *Ctx-M-1* and *shv* genes were detected at varying frequencies in different antibiotic resistance profiles, although the *p*-values were consistently higher than 0.05, indicating no significant correlation.

### 3.6. Antibiotic Resistance Gene Prevalence

**Figure 2:**
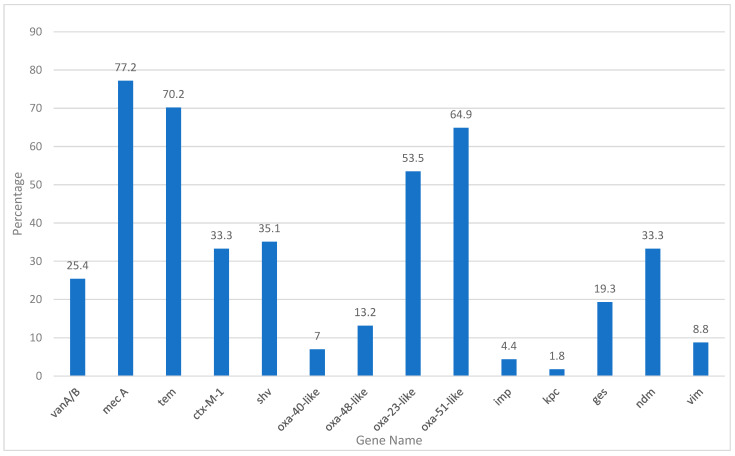
Antibiotic resistance genes’ percentage.

In this study, the prevalence of 14 antibiotic resistance genes was evaluated in the tested samples. Mec A was the most commonly detected gene, identified in 87 samples, corresponding to 77.2% of the total samples analyzed (Table 14 and Figure 2). This gene, known to confer resistance to methicillin and other beta-lactam antibiotics, reflects the significant presence of methicillin-resistant *Staphylococcus aureus* (MRSA) or other resistant strains. Similarly, the *Tem* gene, which is associated with extended-spectrum beta-lactamases (ESBLs), was detected in 80 samples (70.2%), indicating beta-lactam resistance (Table 14 and Figure 2).

Other notable findings include the *Oxa-51-like* and *Oxa-23-like* genes detected in 71 (64.9%) and 61 (53.5%) samples, respectively. These genes are typically linked to carbapenem resistance in *Acinetobacter baumannii* and other Gram-negative pathogens. Their high prevalence signals a significant threat posed by carbapenem-resistant organisms in the tested population. Furthermore, the *Ctx-M-1* gene, another key marker for ESBL production, was identified in 38 samples (33.3%), underscoring the continued prevalence of β-lactamase-producing organisms.

Genes associated with other forms of resistance, such as *shv* and *Ndm*, were also frequently detected. The *shv* gene, which codes for beta-lactamases, was identified in 40 samples (35.1%), whereas the *Ndm* gene, responsible for carbapenemase production, was present in 38 samples (33.3%) (Table 14 and Figure 2). These findings highlight the growing challenge of multidrug-resistant pathogens, particularly those that produce carbapenemases and ESBLs.

The detection of *VanA/B* (29 samples, 25.4%), *ges* (22 samples, 19.3%), and *Oxa-48-like* bacteria (15 samples, 13.2%) further emphasized the presence of resistance mechanisms in the population. The *ges* gene, which is associated with resistance to aminoglycosides, and *Oxa-48-like*, a carbapenemase gene, further highlight the diverse range of resistance mechanisms present in the tested samples.

Lower frequencies of detection were observed for *Oxa-40-like* (7%), *Vim* (8.8%), and *Imp* (4.4%) genes. *Oxa-40-like* and *Vim* genes, both related to carbapenem resistance, were detected in a smaller proportion of samples; however, their presence indicates potential concerns in the resistance landscape. The *imp* gene, found in only five samples (4.4%), also contributes to carbapenem resistance, although it appears to be less prevalent in this study. The *Kpc* gene, which is associated with carbapenemase production, was the least commonly detected, appearing in only two samples (1.8%) (Table 14 and Figure 2).

The distribution of these resistance genes demonstrated a complex and multifaceted resistance profile within the population. The high prevalence of *Mec A*, *Tem*, and *shv* suggests a substantial burden of β-lactam and methicillin resistance. Meanwhile, the presence of carbapenemase genes, such as *Oxa-51-like*, *Oxa-23-like*, *Ndm*, and *Kpc*, emphasizes the growing threat of multidrug-resistant, Gram-negative bacteria. Additionally, the detection of *VanA/B* and ges highlights the ongoing issue of glycopeptide and aminoglycoside resistance.

### 3.7. Comparison Between Culture and qPCR

**Figure 3:**
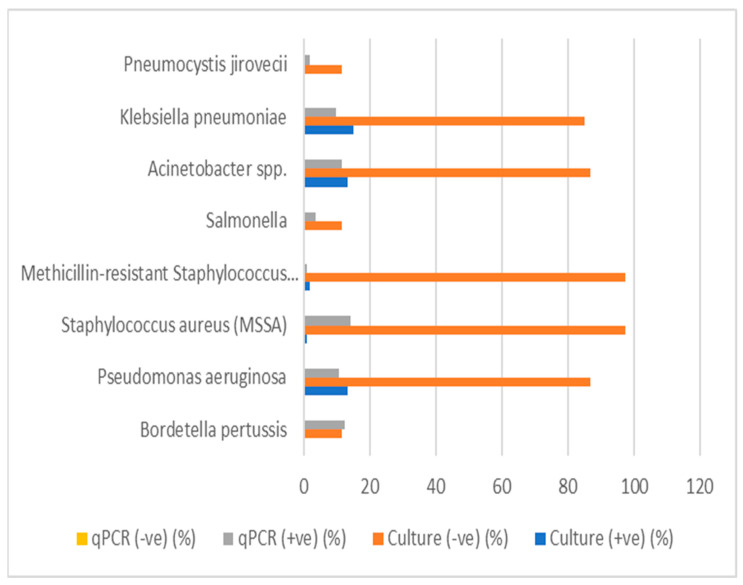
Comparison between culture and qPCR detection of respiratory pathogens.

A comparison of culture and qPCR detection of various respiratory pathogens was conducted to assess the diagnostic performance of both methods. The results for each pathogen are summarized in Table 15 and Figure 3. The table includes valid percentages for positive and negative culture results, qPCR results, and *p*-values for statistical significance.

*Bordetella pertussis*: A low percentage (12.3%) of positive results were detected using qPCR, with no culture-positive samples. The *p*-value (0.152) indicated no statistically significant difference between the culture and qPCR results for this pathogen.

A number of pathogens, including *Streptococcus pneumoniae*, *Mycoplasma pneumoniae*, non-typeable *Haemophilus influenzae*, *Moraxella catarrhalis*, *Chlamydia pneumoniae*, and *Legionella pneumophila*, showed a 100% negative culture rate; however, no positive qPCR results were found for most pathogens. The *p*-values were marked as “a”, indicating that no statistical test was conducted or that these pathogens did not meet the testing criteria.

*Pseudomonas aeruginosa:* This pathogen showed 13.2% positive culture results and 10.5% positive qPCR results, with a *p*-value of <0.001 (Table 15), suggesting a highly significant difference between the culture and qPCR detection methods.

Methicillin-sensitive *Staphylococcus aureus* (MSSA) and MRSA: While MSSA and MRSA showed a low percentage of positive culture results (0.9% and 1.8%, respectively), both showed significantly higher qPCR positivity (14% for MSSA and 0.9% for MRSA). The *p*-values were 0.778 for MSSA and <0.001 for MRSA, indicating no significant difference for MSSA, but a significant difference for MRSA.

*Salmonella*, *Acinetobacter spp.*, *Klebsiella pneumoniae: Salmonella* had a culture positivity of 0%; however, qPCR detected 3.5% positive samples, with a *p*-value of 0.465, suggesting no significant difference between the two methods. *Acinetobacter spp.* and *Klebsiella pneumoniae* showed similar findings, with culture positivity percentages of 13.2% and 14.9%, respectively, and qPCR positivity of 11.4% and 9.6%, respectively. Both had *p*-values of <0.001, indicating no significant differences.

*Legionella longbeachae:* This pathogen showed no percentage of positive culture (0%) and no qPCR positivity (0%), with all results marked as “not available” for qPCR.

*Pneumocystis jirovecii:* This pathogen showed only qPCR positivity of 1.8%.

## 4. Discussion

This study used PCR to detect antibiotic resistance genes in pneumonia pathogens, providing rapid and precise identification of genetic resistance mechanisms. These findings shed light on the genetic basis of antibiotic resistance in patients with pneumonia, highlighting current management challenges. Empirical antibiotic treatment poses significant challenges, particularly in low- and middle-income countries with widespread multidrug-resistant bacteria [14]. Rapid molecular PCR-based diagnostics are urgently needed to guide targeted antibiotic therapy and mitigate delayed treatment, mortality, and antimicrobial resistance [15,16].

This study used a multiplex PCR assay for the rapid detection of 14 antibiotic resistance genes directly from respiratory samples to facilitate timely diagnosis and targeted therapy. Our study identified the *Mec A gene* in 77.2% of the samples, making it the most prevalent resistance gene detected. This finding corroborates the global evidence underscoring *Mec A’s* critical role in methicillin resistance, particularly among *Staphylococcus aureus* isolates. The strong association between *Mec A* and methicillin resistance (*p* < 0.001) emphasizes its clinical significance in managing *S. aureus*-related infections, including pneumonia [17].

Carbapenem resistance genes (*Oxa-48-like*, *Ndm*, *Kpc*) exhibited varying prevalence rates, with *Oxa-48-like* detected in 13.1% of samples. Although statistical significance was not achieved (*p* > 0.05), these genes highlight the emerging threat of carbapenemase-producing pathogens in multidrug-resistant pneumonia [18].

ESBL genes, specifically *Ctx-M-1* and *Shv*, have confirmed the prevalence of beta-lactam resistance. Notably, *Ctx-M-1* (33.3%) contributes significantly to global cephalosporin resistance, emphasizing the importance of considering ESBL-producing bacteria in empirical pneumonia treatment [19]

The *Oxa-51-like* gene demonstrated a borderline significant association with ertapenem resistance (*p* = 0.046), suggesting its potential role in conferring resistance. Typically intrinsic to *Acinetobacter baumannii* [18], this finding underscores the need for targeted surveillance of *A. baumannii* infections, particularly in mechanically ventilated patients.

The hospital selected antibiotics, such as meropenem and piperacillin–tazobactam, primarily for their broad-spectrum efficacy against various pathogens, particularly those causing severe infections. Meropenem has emerged as the most frequently used empirical antibiotic because of its effectiveness against resistant Gram-negative bacteria. In contrast, piperacillin–tazobactam was chosen because of its synergistic effects and ability to cover both Gram-positive and Gram-negative organisms.

However, the widespread use of these antibiotics poses the risk of developing resistance. Notably, the prevalence of resistance genes, such as *Oxa-51-like* and *Oxa-23-like*, signifies a considerable threat to carbapenem-resistant organisms. Resistance rates for carbapenems were concerning, with 23.7% for imipenem and 24.6% for meropenem; similar resistance was observed with piperacillin–tazobactam at 30.7% [20].

Discrepancies arise when sensitivity tests are compared with antibiotic utilization. For example, although the sensitivity to meropenem is 30.7%, it is used in 31.6% of cases, indicating that prescriptions often occur despite a significant portion of pathogens exhibiting resistance. Similarly, although piperacillin–tazobactam has a low sensitivity (21.1%), it is frequently used. This gap stresses the importance of aligning empirical treatment decisions with actual resistance patterns and highlights the necessity for ongoing surveillance and antibiotic stewardship programs to effectively combat resistance development. Given the rising failure rates of conventional antibiotics, there is an urgent need to explore alternative therapeutic strategies. Natural products, with their diverse bioactive compounds, and bacteriophage therapy, which utilizes viruses that specifically target bacteria, represent promising options to combat resistant pathogens [21,22].

The results of this study highlight the significant diagnostic advantages of qPCR over traditional culture-based methods for detecting various respiratory pathogens. For example, qPCR was identified in 12.3% of cases, while no positive results were obtained through culture, although the *p*-value (0.152) was not statistically significant [23]. *Pseudomonas aeruginosa* displayed a significant difference between culture (13.2%) and qPCR (10.5%), with a highly significant *p*-value (<0.001). This emphasizes the ability of qPCR to complement or even surpass culture methods for detecting certain pathogens [24]. Similarly, both MSSA and MRSA showed low culture positivity (0.9% and 1.8%, respectively), but much higher qPCR detection rates (14% for MSSA and 0.9% for MRSA). The *p*-value for MSSA (0.778) indicated no significant difference, while MRSA (*p* < 0.001) highlighted qPCR’s superiority of qPCR in detecting resistant strains. Other pathogens, such as *Salmonella*, *Acinetobacter* spp., and *Klebsiella pneumoniae*, presented with low culture positivity but comparable qPCR detection, with *p*-values suggesting no significant difference for *Salmonella* (0.465) but highly significant differences for *Acinetobacter* spp. and *Klebsiella pneumoniae* (both <0.001). The results of *Pneumocystis jirovecii* indicated the importance of including this fungus in clinical practice [25,26]. Overall, the findings of this study strongly support the integration of qPCR into routine clinical diagnostics [27].

A comparison of culture and PCR methods for detecting bacteria revealed that, in some cases, culture proved more effective than PCR, which is typically expected to be more sensitive. However, errors in performing PCR, such as sample handling, DNA extraction, personnel errors, and contamination, can lead to false-negative results. For example, *Pseudomonas aeruginosa* showed 13.2% culture positivity compared to 10.5% using qPCR. Similarly, for *Staphylococcus aureus* (MRSA), culture positivity was 0.9%, whereas qPCR positivity was notably higher at 14%. In the case of *Acinetobacter* spp., 13.2% were culture-positive and 11.4% were qPCR-positive, while *Klebsiella pneumoniae* showed 14.9% culture positivity and 9.6% qPCR positivity.

PCR offers several advantages over traditional culture-based methods, making it a superior diagnostic tool for many clinical settings. One of the key benefits of PCR is its high sensitivity, which allows the detection of low levels of pathogens that may go undetected with culture methods. Additionally, PCR has high specificity, enabling the precise identification of particular DNA sequences from complex mixtures [8,16,28]. This method is also extremely fast compared with traditional techniques, providing rapid results that can significantly expedite clinical decision-making and patient management [29]. Another advantage is that PCR can be performed easily and can use various fluid samples, including blood, without being affected by prior antimicrobial therapy. This minimizes the risk of the emergence of multidrug-resistant pathogens and reduces the need for unnecessarily administered drugs [29]. Furthermore, PCR minimizes the risk of cross-contamination and allows for the simultaneous identification of multiple microorganisms and resistance genes, thus enhancing diagnostic throughput and accuracy. However, it is important to note that PCR also has some limitations, such as higher costs compared to traditional methods, inability to assess susceptibility to all antibiotics, and challenges in diagnosing infections due to non-viable organism detection [16,25]. Despite these drawbacks, the significant advantages of PCR make it an invaluable tool in modern diagnostics, especially in the fight against antibiotic resistance.

Despite the valuable insights provided by this study, several limitations should be considered when interpreting the results. First, the sample size, although adequate for a preliminary analysis, may not fully represent the broader population of patients with pneumonia. A larger cohort across multiple centers is needed to confirm the generalizability of these findings, particularly given the variation in resistance patterns that may exist across different geographical regions.

Second, the study focused primarily on a targeted set of resistance genes, which may not capture the full spectrum of resistance mechanisms present in pneumonia. It is possible that other uncharacterized genes or mechanisms, such as efflux pump systems or alterations in antibiotic targets, contribute to resistance but were not included in the analysis. Additionally, this study did not assess the phenotypic resistance of the pathogens, which would have provided complementary data on the clinical significance of the detected genes.

Furthermore, although PCR is a powerful tool for identifying resistance genes, it does not provide information about the functional expression of these genes in bacterial populations. In some cases, resistance genes may be present but do not actively contribute to the resistance phenotype. Future studies using whole-genome sequencing or transcriptomic approaches could provide a more comprehensive understanding of the genetic and phenotypic relationships between resistance genes and phenotypes.

Another limitation is the use of a single diagnostic kit (Bacresista GLA kit), which may not have detected all possible resistance genes, particularly those that are rare or specific to certain pathogens. Future work could involve using broader or more customized panels for PCR detection to ensure the inclusion of a wider variety of resistance determinants.

## 5. Conclusions

This study comprehensively investigated the prevalence of antibiotic resistance genes in pneumonia pathogens using PCR, with a focus on several key antibiotic classes, including β-lactams and carbapenems. These findings highlight the complex and dynamic nature of antibiotic resistance in pneumonia pathogens and underscore the critical need for robust molecular surveillance to guide treatment decisions and combat the growing threat of resistant infections.

This study also highlighted the advantages of using PCR as a diagnostic tool to detect resistance genes. PCR provides a rapid, sensitive, and specific method for detecting resistance genes directly in clinical samples, including those from patients with negative or slow-growing cultures. The increased sensitivity of PCR over traditional culture techniques was particularly evident in the detection of pathogens, such as *Staphylococcus aureus* (MSSA and MRSA) and *Pseudomonas aeruginosa*, where PCR detected resistance in cases where culture failed to identify. This underscores the potential role of PCR in improving the diagnostic accuracy of respiratory infections, thereby enabling more targeted and timely therapeutic interventions.

### Future Work

Future studies should aim to expand the findings of this research by addressing the limitations outlined above. Key directions for future work include the following.

- Larger Multicenter Studies: A larger and more geographically diverse sample would allow for more robust data on the prevalence and distribution of antibiotic resistance genes across different populations. Multicenter studies could provide a clearer picture of the regional differences in resistance patterns, which is crucial for tailoring local and national antibiotic stewardship policies.
- Broadening the Range of Resistance Genes Studied: Future studies should include a broader range of resistance genes, particularly those related to newer antibiotics and emerging resistance mechanisms. Whole-genome sequencing or next-generation sequencing (NGS) can provide a more comprehensive assessment of both known and novel resistance determinants, including those associated with plasmids, integrons, or other mobile genetic elements. This would offer a more complete understanding of the genetic basis of resistance to pneumonia.
- Phenotypic Resistance Testing: Complementing molecular techniques, such as PCR, with phenotypic resistance testing could help establish a clearer link between gene presence and antibiotic resistance. This would be especially useful for genes that are not fully expressed or that may have reduced functional significance in clinical isolates.
- Functional Studies: Investigating the functional role of resistance genes, such as *Ndm*, *Oxa-48-like*, and *Ctx-M-1*, in clinical isolates would provide valuable insights into how these genes contribute to resistance in a dynamic clinical setting. Transcriptomic analyses or protein assays can help determine the expression levels and activity of these genes under different conditions, thus further clarifying their role in infection outcomes.
- Development of PCR-based Diagnostic Tools for Routine Clinical Use: As this study demonstrates the value of PCR in detecting resistance genes, future research could focus on optimizing and validating PCR-based diagnostic kits for routine clinical use in pneumonia diagnosis. Such kits could be used to rapidly identify both pathogens and their resistance profiles, allowing for faster and more targeted treatment strategies.

## Figures and Tables

**Table 1:** Study framework.

Aspect	Description
Study Type	Observational study
Objective	Identification of antibiotic resistance genes
Year of Study	2022
Study Clinical Site	Islamic Hospital
Sample Size	114 patients and lower respiratory tract samples

**Table 2:** Participant selection criteria.

Category	Inclusion Criteria	Exclusion Criteria
Diagnosis	Clinically confirmed pneumonia	Non-pneumonia cases
Sample Quality	-	Contaminated or insufficient samples
IRB		Patients or their guardians provided informed consent for the use of their samples in this study

**Table 3:** Patient demographics and clinical characteristics.

Category	Parameter
Demographics	Age
	Gender
	Length of Stay (LOS)
	ICU Length of Stay (ICU-LOS)
	Clinical Outcome
	Microbial Pathogenesis
	Antibiotic Utilization (Empirical Initial and Next Therapy)
Clinical Features	Cough
	Shortness of Breath (SOB)
	Fever
	Sputum Production
	Pleuritic Chest Pain
	Hemoptysis or Rust-Colored Sputum
	Respiratory Distress
	Chronic Obstructive Pulmonary Disease (COPD)
	Asthma
	Immunosuppressive Treatment History
	Aspiration Pneumonia
	Patients Requiring Intubation with Mechanical Ventilation
	Prior Antibiotic Use
	Pneumonia Severity Index (PSI)
	CURB-65 Score
Microbiological Findings	Pathogen Identification via PCR and Culture Results

**Table 4:** Reagents/materials and their purposes.

Reagent/Material	Purpose
Lysis Buffer	Breaks cell walls to release DNA
Binding Buffer	Facilitates the binding of DNA to the spin column by providing a suitable environment for DNA adsorption.
Wash Buffers	Washes away contaminants and impurities from the bound DNA. Typically includes ethanol to help in the washing process.
Elution Buffer	Contains a low salt solution that helps elute the DNA from the column into a clean collection tube.
Proteinase K	Breaks down proteins.
Spin Columns	Contains a silica membrane that binds DNA during the binding step and allows for the separation of DNA from contaminants during washing.
Collection Tubes	Collects the flow-through during the binding and washing steps and holds the eluted DNA after the final elution step.

**Table 5:** Materials and methods for DNA extraction.

Step	Material/Method	Description
1. Cell Lysis	ZR BashingBead™ Lysis Tubes, Lysis Solution, Proteinase K	200 µL of biofluid was added to the lysis tubes; 750 µL of Lysis Solution and 20 µL of Proteinase K were added, followed by incubation at 55 °C for 30 min to lyse bacterial cells.
2. Bead Beating	Omni Bead Ruptor Elite^®^, Bashing Bead™ Tubes	Bead-beating conducted for 1 min at 6 m/s, with 5-min rest intervals for 3 cycles to break open bacterial cells.
3. DNA Binding	ZymoBIOMICS™ DNA Binding Buffer, Zymo-Spin™ IICR Column	1200 µL of Binding Buffer was mixed with the lysate and aliquoted into the spin column. Centrifuged at 10,000× *g* for 1 min to bind DNA.
4. Washing	Wash Buffers 1 and 2	Sequential washing with 400 µL of Wash Buffer 1 and 700 µL of Wash Buffer 2, followed by centrifugation at 10,000× *g* for 1 min to remove contaminants.
5. DNA Elution	DNase/RNase-Free Water, Zymo-Spin™ III-HRC Filter, HRC Prep Solution	Elution of DNA was achieved by adding 100 µL of water to the column and centrifuging. DNA was further purified using HRC Prep Solution, and the final elution was conducted at 16,000× *g* for 3 min.

ZR BashingBead™, ZymoBIOMICS™, Zymo-Spin™: Tustin, CA, USA. Omni Bead Ruptor Elite^®^: Osaka, Japan.

**Table 6:** Resistance genes detected using BacResista GLA Kits and their associated antibacterial drugs.

Resistance Gene	Associated Antibacterial Drugs
*VanA/B*	Vancomycin, Teicoplanin
*MecA*	Methicillin, Oxacillin
*Tem*	Penicillins and Cephalosporins
*Ctx-M-1*	
*shv*	
*Oxa-40-like*	Carbapenems
*Oxa-48-like*	
*Oxa-23-like*	
*Oxa-51-like*	
*imp*	
*Kpc*	
*ges*	
*Ndm*	
*Vim*	

**Table 7:** PCR method and reagents.

Step	Reagent/Material	Purpose
1. PCR Reaction Setup	Taq Polymerase	Enzyme responsible for synthesizing DNA strands
	Primers	Short DNA sequences that bind to target DNA regions
	Nucleotide Mix	Provides the building blocks (dNTPs) for DNA synthesis
	PCR Buffer	Maintains the optimal pH and salt conditions for the reaction
Thermal Cycling	Thermal Cycler	A machine that cycles through different temperatures (denaturation, annealing, extension)
3. Amplification	Mineral Oil	Prevents evaporation during the thermal cycling process
4. Controls	Positive Control	Contains known target DNA to ensure the reaction works correctly
	Negative Control	Contains no DNA to check for contamination or false positives

**Table 8:** Detection channels of amplification products.

Tube No.	Dye Label/Detection Channel					Color of the Buffer
	FAM	HEX	ROX	CY5	CY5.5	
Bacresista GLA						
1	*Imp*	IC **	-	-	-	Blue
2	TBL *	IC	-	*Oxa-51-like*	-	Colorless
3	*Ctx-M-1*		-	*Tem*	-	
4	*Van A/B*	IC	-	*Mec A*	-	
5	*Oxa-48-like*	IC	-	*Oxa-40-like*	-	
6	*Vim*	IC	-	*Kpc*	-	
7	*Oxa-23-like*	IC	-	*Ndm*	-	
8	*shv*	IC	Marker	*ges*	-	

* TBL: Total bacterial load. ** IC: Internal control.

**Table 9:** PCR amplification program.

Stage	Step №	Temperature, °C	Time min:s	Number of Cycles (Repeats)
Hold Stage	1	80	01:00	1
	2	94	01:30	1
PCR Stage	1	94	0:20	50
	2	64	0:25	

**Table 10:** Summary of demographic, clinical, microbial, and treatment characteristics of pneumonia patients.

Category	Variable	Value	Details
Demographics	Total Patients	114	-
	Median Age	73 years	Mean: 64.114 years (±20.331)
	Gender Distribution	Male: 63 (55.263%)	Female: 51 (44.737%)
Length of Stay (LOS)	Median LOS	7 days	Mean: 11.272 days (±15.091)
	ICU LOS	Median: 1 day	Mean: 4.763 days (±11.882)
Hospitalization History	Median No. of Hospitalizations	2 day	Mean: 2.772 (±3.005)
	Median No. of Pneumonia Episodes	0.5 day	Mean: 0.895 (±1.366)
Outcomes	Mortality Rate	27 patients (23.684%)	Improved: 87 (76.316%)
	CAP Prevalence	100%	All cases were community-acquired pneumonia
Microbial Pathogenesis	Bacterial Infections	33 (28.947%)	-
	Bacterial–Viral Co-infections	28 (24.561%)	-
	Viral Infections	23 (20.175%)	-
	Polybacterial Infections	12 (10.526%)	-
	Polymicrobial Viral Infections	15 (13.158%)	-
	Fungal-Bacterial Co-infections	2 (1.754%)	-
Sample Types	Sputum (SPU)	102 (89.474%)	Predominant diagnostic sample type
	Fluid Samples	5 (4.386%)	-
	BAL	3 (2.632%)	Bronchoalveolar lavage
	Endotracheal Aspirates	3 (2.632%)	-
	Nasopharyngeal Swabs (NAS)	1 (0.877%)	-
Clinical Features	Shortness of Breath (SOB)	56 (49.123%)	Most common presenting symptom
	Cough	15 (13.158%)	-
	Fever	14 (12.281%)	-
	Sputum Production	7 (6.140%)	-
	Pleuritic Chest Pain	9 (8.738%)	-
	Hemoptysis/Rust-colored Sputum	11 (9.649%)	-
Severity Scores	CURB-65 Scores	1: 66 (57.895%), 2: 20 (17.544%)	Score 1 reflects low risk; score 2 reflects moderate risk
	PSI (Pneumonia Severity Index)	-	Range: 8–150
Treatment Adherence	Guideline-Concordant Antibiotics	33 (35.106%)	-
	Completion of Antibiotic Therapy	72 (63.158%)	Incomplete in 42 cases (36.842%)

**Table 11:** Pneumonia severity index (PSI) classification, mortality rates, and guideline-concordant antibiotic therapy.

Category	Class I (Low Risk, PSI ≤ 70)	Class II (Low to Moderate Risk, PSI 71–90)	Class III (Moderate Risk, PSI 91–130)	Class IV (High Risk, PSI > 130)
PSI Score Range	≤70	71–90	91–130	>130
Mortality Rate	0.1%	0.6%	0.9%	9.3%
Number of Patients	59.649% (n = 68)	24.561% (n = 28)	8.772% (n = 10)	7.018% (n = 8)
Description	Low risk, typically managed with outpatient care.	Low to moderate risk, outpatient care or observation.	Moderate risk, typically requiring inpatient admission.	High risk, requires inpatient admission due to elevated mortality.
Guideline-Concordant Antibiotic Therapy	Use antibiotics recommended for typical pathogens and empirical therapy, adjusted once pathogen is identified.	Empiric therapy based on likely pathogens, considering local resistance.	Inpatient treatment based on the patient’s risk and local resistance patterns.	High-risk patients are treated aggressively with appropriate antibiotics based on severity and resistance patterns.

**Table 12:** Antibiotic prescription patterns.

Category	Antibiotic	Number of Patients (n)	Percentage (%)	Details
Empiric Therapy	Meropenem	36	31.60%	Most frequently used for severe infections.
	Piperacillin–tazobactam	30	26.30%	Broad-spectrum agent for Gram-negative and polymicrobial infections.
	Levofloxacin	4	3.50%	Targets respiratory pathogens.
	Amoxicillin–clavulanate	9	7.90%	Used for moderate infections, particularly when ESBL-producing organisms are less likely.
	Ceftriaxone	7	6.10%	Commonly prescribed for community-acquired infections.
	Doxycycline	1	0.90%	Targeted use for atypical pathogens.
	Amikacin	2	1.80%	Used for Gram-negative organisms, typically in severe infections.
	Ampicillin	4	3.50%	Commonly prescribed for less severe Gram-positive infections.
	Azithromycin	5	4.40%	Often used for atypical respiratory pathogens.
	Vancomycin	27	23.70%	Used for suspected MRSA infections.
Next-Line Therapy	Amoxicillin–clavulanate	16	14.04%	Continued when pathogens are susceptible, representing a shift to narrow-spectrum therapy.
	Azithromycin	11	9.64%	Often used for respiratory infections or atypical coverage.
	Colistin	11	9.64%	Escalated for multidrug-resistant Gram-negative pathogens.
Initial Continuation Therapy	Ceftriaxone	1	0.88%	Continued when culture results support its efficacy.
	Amoxicillin–clavulanate	1	0.88%	Continued when pathogens are susceptible to narrow-spectrum therapy.
	Colistin	13	11.40%	Used for multidrug-resistant Gram-negative organisms.
	Amikacin	5	4.39%	Continued for Gram-negative coverage in severe cases.
	Metronidazole	2	1.75%	Used for anaerobic infections, particularly in intra-abdominal or polymicrobial infections.
	Levofloxacin	3	2.63%	Targets respiratory pathogens.
	Doxycycline	8	7.02%	Continued for atypical pathogen coverage.
	Azithromycin	7	6.14%	Often used for respiratory infections or atypical coverage.

**Table 13:** Association between antibiotic resistance genes and antibiotic susceptibility.

Antibiotic	Gene Name	Resistance Gene (%)	Susceptibility (Frequency)		*p*-Value (Chi-Square)
			Resistance	Sensitive	
Imipenem	*Oxa-40-like*	7	2	2	0.915
	*Oxa-48-like*	13.2	6	5	0.200
	*Oxa-23-like*	53.5	17	19	0.499
	*Oxa-51-like*	64.9	22	24	0.062
	*Imp*	4.4	1	0	0.209
	*Kpc*	1.8	1	0	0.535
	*Ndm*	33.3	11	12	0.601
	*Vim*	8.8	3	0	0.074
Meropenem	*Oxa-40-like*	6	2	2	0.930
	*Oxa-48-like*	13.2	6	5	0.226
	*Oxa-23-like*	53.5	18	18	0.410
	*Oxa-51-like*	64.9	22	24	0.093
	*Imp*	4.4	1	0	0.212
	*Kpc*	1.8	1	0	0.556
	*Ndm*	33.3	11	12	0.666
	*Vim*	8.8	3	0	0.080
Etrapenem	*Oxa-40-like*	7	2	1	0.811
	*Oxa-48-like*	13.2	5	2	0.357
	*Oxa-23-like*	53.5	16	10	0.151
	*Oxa-51-like*	64.9	20	14	0.046
	*Imp*	4.4	1	0	0.472
	*Kpc*	1.8	1	0	0.508
	*Ndm*	33.3	9	6	0.569
	*Vim*	8.8	3	0	0.216
Pipracillin-Tazobactam	*Tem*	70.2	23	17	0.904
	*Ctx-M-1*	33.3	14	6	0.692
	*Shv*	35.1	13	11	0.249
Cefepime	*Tem*	70.2	12	11	0.848
	*Ctx-M-1*	33.3	13	6	0.553
	*shv*	35.1	12	12	0.417
Ceftriaxone	*Tem*	70.2	14	15	0.656
	*Ctx-M-1*	33.3	12	5	0.431
	*Shv*	35.1	12	8	0.375
Amoxicillin-Clavulanic Acid	*Tem*	70.2	24	9	0.888
	*Ctx-M-1*	33.3	13	3	0.413
	*shv*	35.1	14	5	0.329
Ceftazidime	*Tem*	70.2	23	19	0.762
	*Ctx-M-1*	33.3	15	6	0.230
	*shv*	35.1	14	12	0.231
Cefotaxime	*Tem*	70.2	19	15	0.656
	*Ctx-M-1*	33.3	12	5	0.431
	*shv*	35.1	12	8	0.375
Cefpodoxime	*Tem*	70.2	21	13	0.577
	*Ctx-M-1*	33.3	12	5	0.758
	*shv*	35.1	12	8	0.280
Cefixime	*Tem*	70.2	25	8	0.776
	*Ctx-M-1*	33.3	14	3	0.693
	*shv*	35.1	16	4	0.294
Cefuroxime	*Tem*	70.2	26	7	0.867
	*Ctx-M-1*	33.3	14	3	0.770
	*shv*	35.1	17	3	0.236
Cefazolin	*Tem*	70.2	26	8	0.765
	*Ctx-M-1*	33.3	14	3	0.770
	*shv*	35.1	17	3	0.242
Ampicillin	*Tem*	70.2	31	2	0.641
	*Ctx-M-1*	33.3	17	0	0.419
	*shv*	35.1	20	0	0.128
Methicillin	*Mec A*	76.3	1	0	<0.001
Teicoplanin	*VanA/B*	25.4	0	0	0.405

**Table 14:** Antibiotic resistance genes’ frequency.

Gene	Frequency
*VanA/B*	29
*Mec A*	87
*Tem*	80
*Ctx-M-1*	38
*shv*	40
*Oxa-40-like*	8
*Oxa-48-like*	15
*Oxa-23-like*	61
*Oxa-51-like*	71
*Imp*	5
*Kpc*	2
*ges*	22
*Ndm*	38
*Vim*	10

**Table 15:** Comparison of culture and qPCR results for respiratory pathogens.

Bacteria Name	Culture (+ve) (%)	Culture (−ve) (%)	Culture—Not Available (%)	qPCR (+ve) (%)	qPCR (−ve) (%)	qPCR—Not Available (%)	*p*-Value
*Bordetella pertussis*	0	11.4	88.6	12.3	0	87.7	0.152
*Streptococcus pneumoniae*	0	100	0	0	0	100	a
*Mycoplasma pneumoniae*	0	100	0	0.9	0	99.1	a
*Non-typeable Haemophilus influenzae*	0	100	0	2.6	0	97.4	a
*Moraxella catarrhalis*	0	100	0	0	0	100	a
*Chlamydia pneumoniae*	0	100	0	0	0	100	a
*Legionella pneumophila*	0	100	0	0	0	100	a
*Pseudomonas aeruginosa*	13.2	86.8	0	10.5	0	89.5	<0.001
*Staphylococcus aureus (MSSA)*	0.9	97.4	1.8	14	0	86	0.778
*Methicillin-resistant Staphylococcus aureus (MRSA)*	1.8	97.4	0.9	0.9	0	99.1	<0.001
*Salmonella*	0	11.4	88.6	3.5	0	96.5	0.465
*Acinetobacter spp.*	13.2	86.8	0	11.4	0	88.6	<0.001
*Klebsiella pneumoniae*	14.9	85.1	0	9.6	0	90.4	<0.001
*Legionella longbeachae*	0	7.9	92.1	0	0	100	a
*Pneumocystis jirovecii*	0	11.4	88.6	1.8	0	98.2	0.609

“a” indicates that no available statistical test was performed or that these pathogens did not meet the criteria for testing.

## Data Availability

Available upon reasonable request.
